# Dibromido[(*S*)-2-(pyrrolidin-2-yl)-1*H*-benzimidazole]zinc(II)

**DOI:** 10.1107/S1600536808019168

**Published:** 2008-07-09

**Authors:** Wei Dai, Da-Wei Fu

**Affiliations:** a Ordered Matter Science Research Center, College of Chemistry and Chemical Engineering, Southeast University, Nanjing 210096, People’s Republic of China

## Abstract

The title compound, [ZnBr_2_(C_11_H_13_N_3_)], was synthesized by hydro­thermal reaction of ZnBr_2_ and (*S*)-2-(pyrrolidin-2-yl)-1*H*-benzimidazole. The Zn^II^ atom has a distorted tetra­hedral geometry and is coordinated by two N atoms from the chelating organic ligand and two terminal Br^−^ anions. In the crystal structure, mol­ecules are linked into a chain along the [101] direction by N—H⋯Br and C—H⋯Br hydrogen bonds.

## Related literature

For physical properties such as fluorescence and dielectric behaviors of metal-organic coordination compounds, see: Aminabhavi *et al.* (1986 ▶); Ye *et al.* (2008 ▶); Fu *et al.* (2007 ▶).
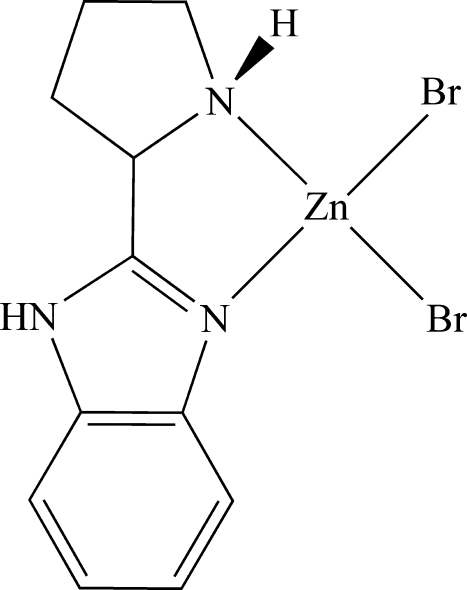


## Experimental

### 

#### Crystal data

[ZnBr_2_(C_11_H_13_N_3_)]
*M* *_r_* = 412.43
Monoclinic, 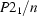

*a* = 8.953 (3) Å
*b* = 11.668 (2) Å
*c* = 13.318 (2) Å
β = 91.443 (3)°
*V* = 1390.9 (6) Å^3^
*Z* = 4
Mo *K*α radiation
μ = 7.49 mm^−1^
*T* = 298 (2) K
0.30 × 0.25 × 0.15 mm

#### Data collection

Rigaku Mercury2 diffractometer
Absorption correction: multi-scan (*CrystalClear*; Rigaku, 2005 ▶) *T*_min_ = 0.459, *T*_max_ = 0.982 (expected range = 0.152–0.325)
13896 measured reflections
3179 independent reflections
2426 reflections with *I* > 2σ(*I*)
*R*_int_ = 0.065

#### Refinement

*R*[*F* ^2^ > 2σ(*F* ^2^)] = 0.047
*wR*(*F* ^2^) = 0.114
*S* = 0.99
3179 reflections
154 parameters
H-atom parameters constrained
Δρ_max_ = 0.67 e Å^−3^
Δρ_min_ = −1.00 e Å^−3^

### 

Data collection: *CrystalClear* (Rigaku, 2005 ▶); cell refinement: *CrystalClear*; data reduction: *CrystalClear*; program(s) used to solve structure: *SHELXS97* (Sheldrick, 2008 ▶); program(s) used to refine structure: *SHELXL97* (Sheldrick, 2008 ▶); molecular graphics: *SHELXTL* (Sheldrick, 2008 ▶); software used to prepare material for publication: *SHELXL97*.

## Supplementary Material

Crystal structure: contains datablocks I, global. DOI: 10.1107/S1600536808019168/ci2621sup1.cif

Structure factors: contains datablocks I. DOI: 10.1107/S1600536808019168/ci2621Isup2.hkl

Additional supplementary materials:  crystallographic information; 3D view; checkCIF report

## Figures and Tables

**Table:** 

Br1—Zn1	2.3642 (8)
Zn1—N2	2.011 (3)
Zn1—N1	2.075 (4)
Zn1—Br2	2.3319 (7)

**Table:** 

N2—Zn1—N1	82.35 (14)
N2—Zn1—Br2	112.70 (10)
N1—Zn1—Br2	117.89 (10)
N2—Zn1—Br1	118.99 (11)
N1—Zn1—Br1	110.08 (11)
Br2—Zn1—Br1	112.03 (3)

**Table 2:** Hydrogen-bond geometry (Å, °)

*D*—H⋯*A*	*D*—H	H⋯*A*	*D*⋯*A*	*D*—H⋯*A*
N3—H3*C*⋯Br1^i^	0.86	2.74	3.516 (4)	150
C4—H4*A*⋯Br1^ii^	0.98	2.86	3.637 (5)	137
C1—H1*A*⋯*Cg*1^iii^	0.97	2.78	3.673 (6)	153

Symmetry codes: (i) 
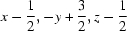
; (ii) 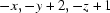; (iii) 
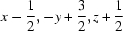
. *Cg*1 is the centroid of the C6–C11 ring.

**Fig. 1.**
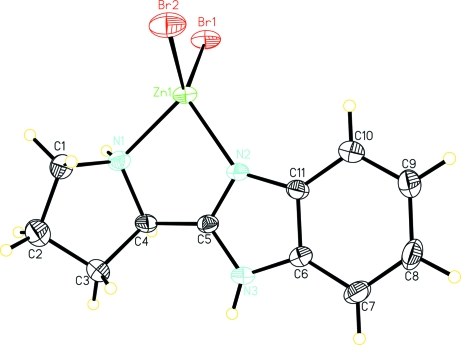
A view of the title compound with the atomic numbering scheme. Displacement ellipsoids are drawn at the 30% probability level.

**Fig. 2.**
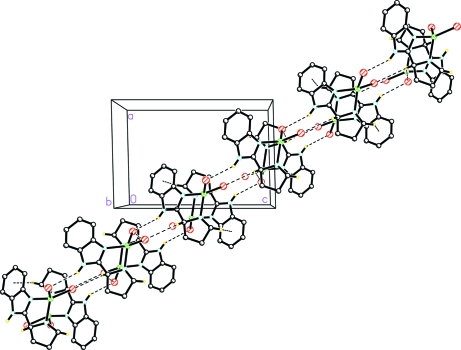
The crystal packing of the title compound, viewed along the b axis. H atoms not involved in hydrogen bonding (dashed lines) have been omitted for clarity.

## supplementary crystallographic information

### Comment

Metal-organic coordination compounds provide a class of complexes displaying interesting chemical and physical properties such as fluorescence and dielectric behaviors (Aminabhavi *et al.*, 1986; Ye *et al.*, 2008; Fu *et al.*, 2007). There has been very strong interest in employing crystal-engineering strategies to generate desirable materials by the hydrothermal reaction. Here we report the synthesis and crystal structure of the title compound.

The Zn^II^ atom has a distorted tetrahedral geometry (Table 1) and is coordinated by two N atoms from the chelating *S*-2-(pyrrolidin-2-yl)-1*H*-benzimidazole ligand and two terminal Br^-^ anions (Fig. 1).

In the crystal structure, N—H···Br and C—H···Br hydrogen bonds (Table 2) link the molecules into a chain along [1 0 1] (Fig.2).

### Experimental

The homochiral ligand *S*-2-(pyrrolidin-2-yl)-1*H*-benzimidazole was synthesized by reaction of *S*-pyrrolidine-2-carboxylic acid and benzene-1,2-diamine according to the procedure described in the literature (Aminabhavi *et al.*, 1986). A mixture of *S*-2-(pyrrolidin-2-yl)-1*H*-benzimidazole (18.7 mg, 0.1 mmol), ZnBr_2_ (33.9 mg, 0.1 mmol) and water (1 ml) sealed in a glass tube was maintained at 343 K. Crystals suitable for X-ray ananlysis were obtained after 3 d.

### Refinement

All H atoms attached to C and N atoms were fixed geometrically and treated as riding with C-H = 0.93 Å (aromatic), 0.97 Å (methylene) or 0.98 Å (methine) and N-H = 0.91 Å with *U*_iso_(H) = 1.2*U*_eq_(C,N).

### Crystal data

**Table:**

[ZnBr_2_(C_11_H_13_N_3_)]	*F*_000_ = 800
*M**_r_* = 412.43	*D*_x_ = 1.970 Mg m^−^^3^
Monoclinic, *P*2_1_/*n*	Mo *K*α radiation λ = 0.71073 Å
Hall symbol: -P 2yn	Cell parameters from 3615 reflections
*a* = 8.953 (3) Å	θ = 2.7–27.5º
*b* = 11.668 (2) Å	µ = 7.49 mm^−^^1^
*c* = 13.318 (2) Å	*T* = 298 (2) K
β = 91.443 (3)º	Block, colourless
*V* = 1390.9 (6) Å^3^	0.30 × 0.25 × 0.15 mm
*Z* = 4	

### Data collection

**Table:**

Rigaku Mercury2 diffractometer	3179 independent reflections
Radiation source: fine-focus sealed tube	2426 reflections with *I* > 2σ(*I*)
Monochromator: graphite	*R*_int_ = 0.065
Detector resolution: 13.6612 pixels mm^-1^	θ_max_ = 27.5º
*T* = 298(2) K	θ_min_ = 2.7º
ω scans	*h* = −11→11
Absorption correction: multi-scan(CrystalClear; Rigaku, 2005)	*k* = −15→15
*T*_min_ = 0.459, *T*_max_ = 0.982	*l* = −17→17
13896 measured reflections	

### Refinement

**Table:**

Refinement on *F*^2^	Secondary atom site location: difference Fourier map
Least-squares matrix: full	Hydrogen site location: inferred from neighbouring sites
*R*[*F*^2^ > 2σ(*F*^2^)] = 0.047	H-atom parameters constrained
*wR*(*F*^2^) = 0.114	*w* = 1/[σ^2^(*F*_o_^2^) + (0.0563*P*)^2^] where *P* = (*F*_o_^2^ + 2*F*_c_^2^)/3
*S* = 0.99	(Δ/σ)_max_ = 0.001
3179 reflections	Δρ_max_ = 0.67 e Å^−^^3^
154 parameters	Δρ_min_ = −1.00 e Å^−^^3^
Primary atom site location: structure-invariant direct methods	Extinction correction: none

### Special details

**Table:**

Geometry. All e.s.d.'s (except the e.s.d. in the dihedral angle between two l.s. planes) are estimated using the full covariance matrix. The cell e.s.d.'s are taken into account individually in the estimation of e.s.d.'s in distances, angles and torsion angles; correlations between e.s.d.'s in cell parameters are only used when they are defined by crystal symmetry. An approximate (isotropic) treatment of cell e.s.d.'s is used for estimating e.s.d.'s involving l.s. planes.
Refinement. Refinement of *F*^2^ against ALL reflections. The weighted *R*-factor *wR* and goodness of fit *S* are based on *F*^2^, conventional *R*-factors *R* are based on *F*, with *F* set to zero for negative *F*^2^. The threshold expression of *F*^2^ > σ(*F*^2^) is used only for calculating *R*-factors(gt) *etc*. and is not relevant to the choice of reflections for refinement. *R*-factors based on *F*^2^ are statistically about twice as large as those based on *F*, and *R*- factors based on ALL data will be even larger.

### Fractional atomic coordinates and isotropic or equivalent isotropic displacement parameters (Å^2^)

**Table:**

	*x*	*y*	*z*	*U*_iso_*/*U*_eq_	
Br1	0.25768 (6)	0.93780 (4)	0.57154 (4)	0.04999 (18)	
Zn1	0.13105 (6)	0.76113 (4)	0.55176 (4)	0.03634 (16)	
Br2	0.20151 (7)	0.62945 (5)	0.67586 (4)	0.0600 (2)	
C6	0.1068 (5)	0.5895 (4)	0.2708 (3)	0.0378 (10)	
N2	0.1146 (4)	0.6901 (3)	0.4143 (2)	0.0344 (8)	
N1	−0.0955 (4)	0.7895 (3)	0.5261 (3)	0.0378 (8)	
H10B	−0.1140	0.8650	0.5377	0.045*	
N3	−0.0296 (4)	0.6401 (3)	0.2863 (3)	0.0391 (9)	
H3C	−0.1073	0.6357	0.2471	0.047*	
C8	0.3041 (6)	0.4841 (5)	0.2047 (4)	0.0554 (14)	
H8A	0.3427	0.4362	0.1559	0.066*	
C5	−0.0198 (5)	0.6977 (4)	0.3735 (3)	0.0315 (9)	
C11	0.1978 (5)	0.6223 (4)	0.3510 (3)	0.0354 (9)	
C7	0.1572 (6)	0.5177 (4)	0.1952 (3)	0.0489 (12)	
H7A	0.0955	0.4941	0.1419	0.059*	
C3	−0.2915 (5)	0.6980 (5)	0.4237 (4)	0.0550 (13)	
H3A	−0.3632	0.7284	0.3748	0.066*	
H3B	−0.2771	0.6170	0.4107	0.066*	
C10	0.3458 (6)	0.5868 (5)	0.3582 (4)	0.0508 (12)	
H10A	0.4074	0.6090	0.4120	0.061*	
C9	0.3976 (6)	0.5188 (5)	0.2844 (4)	0.0555 (14)	
H9A	0.4967	0.4949	0.2869	0.067*	
C1	−0.1969 (6)	0.7209 (5)	0.5893 (3)	0.0512 (13)	
H1A	−0.2104	0.7573	0.6539	0.061*	
H1B	−0.1578	0.6442	0.6002	0.061*	
C4	−0.1416 (5)	0.7637 (4)	0.4201 (3)	0.0361 (10)	
H4A	−0.1568	0.8357	0.3834	0.043*	
C2	−0.3426 (6)	0.7174 (6)	0.5290 (4)	0.0631 (15)	
H2A	−0.4059	0.6553	0.5510	0.076*	
H2B	−0.3965	0.7892	0.5344	0.076*	

### Atomic displacement parameters (Å^2^)

**Table:**

	*U*^11^	*U*^22^	*U*^33^	*U*^12^	*U*^13^	*U*^23^
Br1	0.0629 (3)	0.0386 (3)	0.0474 (3)	−0.0098 (2)	−0.0209 (2)	0.0027 (2)
Zn1	0.0395 (3)	0.0392 (3)	0.0299 (3)	−0.0022 (2)	−0.0093 (2)	−0.0028 (2)
Br2	0.0757 (4)	0.0521 (3)	0.0509 (3)	−0.0016 (3)	−0.0220 (3)	0.0139 (2)
C6	0.040 (2)	0.042 (2)	0.032 (2)	−0.001 (2)	−0.0023 (19)	−0.0055 (18)
N2	0.0326 (18)	0.041 (2)	0.0290 (17)	−0.0002 (16)	−0.0077 (15)	−0.0026 (15)
N1	0.041 (2)	0.0366 (19)	0.0357 (19)	−0.0010 (17)	−0.0034 (16)	−0.0067 (16)
N3	0.035 (2)	0.050 (2)	0.0319 (18)	−0.0031 (17)	−0.0084 (16)	−0.0051 (16)
C8	0.057 (3)	0.063 (3)	0.046 (3)	0.018 (3)	0.014 (3)	−0.013 (3)
C5	0.034 (2)	0.035 (2)	0.0255 (19)	−0.0055 (18)	−0.0032 (17)	0.0030 (17)
C11	0.031 (2)	0.041 (2)	0.033 (2)	−0.0023 (19)	−0.0051 (18)	−0.0002 (18)
C7	0.058 (3)	0.054 (3)	0.035 (2)	0.001 (3)	−0.004 (2)	−0.012 (2)
C3	0.035 (3)	0.077 (4)	0.053 (3)	−0.008 (3)	0.002 (2)	−0.015 (3)
C10	0.039 (3)	0.067 (3)	0.046 (3)	0.002 (3)	−0.008 (2)	−0.010 (2)
C9	0.041 (3)	0.076 (4)	0.050 (3)	0.014 (3)	0.004 (2)	−0.006 (3)
C1	0.048 (3)	0.068 (3)	0.038 (3)	−0.009 (3)	0.004 (2)	0.004 (2)
C4	0.033 (2)	0.044 (3)	0.031 (2)	0.0022 (19)	−0.0080 (18)	0.0031 (18)
C2	0.046 (3)	0.087 (4)	0.057 (3)	−0.002 (3)	0.002 (3)	0.004 (3)

### Geometric parameters (Å, °)

**Table:**

Br1—Zn1	2.3642 (8)	C5—C4	1.484 (6)
Zn1—N2	2.011 (3)	C11—C10	1.390 (6)
Zn1—N1	2.075 (4)	C7—H7A	0.93
Zn1—Br2	2.3319 (7)	C3—C2	1.504 (7)
C6—N3	1.377 (6)	C3—C4	1.547 (6)
C6—C11	1.382 (6)	C3—H3A	0.97
C6—C7	1.393 (6)	C3—H3B	0.97
N2—C5	1.311 (5)	C10—C9	1.354 (7)
N2—C11	1.387 (5)	C10—H10A	0.93
N1—C1	1.488 (6)	C9—H9A	0.93
N1—C4	1.492 (5)	C1—C2	1.515 (7)
N1—H10B	0.91	C1—H1A	0.97
N3—C5	1.343 (5)	C1—H1B	0.97
N3—H3C	0.86	C4—H4A	0.98
C8—C7	1.376 (7)	C2—H2A	0.97
C8—C9	1.395 (7)	C2—H2B	0.97
C8—H8A	0.93		
			
N2—Zn1—N1	82.35 (14)	C6—C7—H7A	122.2
N2—Zn1—Br2	112.70 (10)	C2—C3—C4	103.8 (4)
N1—Zn1—Br2	117.89 (10)	C2—C3—H3A	111.0
N2—Zn1—Br1	118.99 (11)	C4—C3—H3A	111.0
N1—Zn1—Br1	110.08 (11)	C2—C3—H3B	111.0
Br2—Zn1—Br1	112.03 (3)	C4—C3—H3B	111.0
N3—C6—C11	105.8 (4)	H3A—C3—H3B	109.0
N3—C6—C7	132.1 (4)	C9—C10—C11	118.0 (5)
C11—C6—C7	122.0 (4)	C9—C10—H10A	121.0
C5—N2—C11	106.7 (3)	C11—C10—H10A	121.0
C5—N2—Zn1	113.3 (3)	C10—C9—C8	120.8 (5)
C11—N2—Zn1	139.5 (3)	C10—C9—H9A	119.6
C1—N1—C4	105.6 (3)	C8—C9—H9A	119.6
C1—N1—Zn1	115.4 (3)	N1—C1—C2	104.1 (4)
C4—N1—Zn1	111.7 (3)	N1—C1—H1A	110.9
C1—N1—H10B	108.0	C2—C1—H1A	110.9
C4—N1—H10B	108.0	N1—C1—H1B	110.9
Zn1—N1—H10B	108.0	C2—C1—H1B	110.9
C5—N3—C6	107.8 (3)	H1A—C1—H1B	109.0
C5—N3—H3C	126.1	C5—C4—N1	108.1 (3)
C6—N3—H3C	126.1	C5—C4—C3	113.8 (4)
C7—C8—C9	122.8 (5)	N1—C4—C3	106.9 (3)
C7—C8—H8A	118.6	C5—C4—H4A	109.3
C9—C8—H8A	118.6	N1—C4—H4A	109.3
N2—C5—N3	111.4 (4)	C3—C4—H4A	109.3
N2—C5—C4	122.6 (4)	C3—C2—C1	102.7 (4)
N3—C5—C4	126.0 (4)	C3—C2—H2A	111.2
C6—C11—N2	108.2 (4)	C1—C2—H2A	111.2
C6—C11—C10	120.8 (4)	C3—C2—H2B	111.2
N2—C11—C10	130.9 (4)	C1—C2—H2B	111.2
C8—C7—C6	115.6 (4)	H2A—C2—H2B	109.1
C8—C7—H7A	122.2		
			
N1—Zn1—N2—C5	2.2 (3)	Zn1—N2—C11—C6	170.4 (3)
Br2—Zn1—N2—C5	119.3 (3)	C5—N2—C11—C10	−179.8 (5)
Br1—Zn1—N2—C5	−106.5 (3)	Zn1—N2—C11—C10	−9.2 (8)
N1—Zn1—N2—C11	−168.0 (5)	C9—C8—C7—C6	0.1 (8)
Br2—Zn1—N2—C11	−50.9 (5)	N3—C6—C7—C8	179.6 (5)
Br1—Zn1—N2—C11	83.2 (5)	C11—C6—C7—C8	−1.4 (7)
N2—Zn1—N1—C1	110.8 (3)	C6—C11—C10—C9	−0.2 (8)
Br2—Zn1—N1—C1	−0.8 (3)	N2—C11—C10—C9	179.3 (5)
Br1—Zn1—N1—C1	−131.0 (3)	C11—C10—C9—C8	−1.1 (8)
N2—Zn1—N1—C4	−9.8 (3)	C7—C8—C9—C10	1.2 (9)
Br2—Zn1—N1—C4	−121.4 (3)	C4—N1—C1—C2	−32.6 (5)
Br1—Zn1—N1—C4	108.4 (3)	Zn1—N1—C1—C2	−156.5 (3)
C11—C6—N3—C5	−1.6 (5)	N2—C5—C4—N1	−14.3 (6)
C7—C6—N3—C5	177.5 (5)	N3—C5—C4—N1	166.5 (4)
C11—N2—C5—N3	−0.8 (5)	N2—C5—C4—C3	−132.8 (4)
Zn1—N2—C5—N3	−174.2 (3)	N3—C5—C4—C3	47.9 (6)
C11—N2—C5—C4	179.8 (4)	C1—N1—C4—C5	−111.7 (4)
Zn1—N2—C5—C4	6.5 (5)	Zn1—N1—C4—C5	14.4 (4)
C6—N3—C5—N2	1.5 (5)	C1—N1—C4—C3	11.1 (5)
C6—N3—C5—C4	−179.2 (4)	Zn1—N1—C4—C3	137.3 (3)
N3—C6—C11—N2	1.1 (5)	C2—C3—C4—C5	133.9 (4)
C7—C6—C11—N2	−178.1 (4)	C2—C3—C4—N1	14.6 (5)
N3—C6—C11—C10	−179.2 (4)	C4—C3—C2—C1	−34.0 (6)
C7—C6—C11—C10	1.6 (7)	N1—C1—C2—C3	41.8 (6)
C5—N2—C11—C6	−0.2 (5)		

### Hydrogen-bond geometry (Å, °)

**Table:**

*D*—H···*A*	*D*—H	H···*A*	*D*···*A*	*D*—H···*A*
N3—H3C···Br1^i^	0.86	2.74	3.516 (4)	150
C4—H4A···Br1^ii^	0.98	2.86	3.637 (5)	137
C1—H1A···Cg1^iii^	0.97	2.78	3.673 (6)	153

Symmetry codes: (i) *x*−1/2, −*y*+3/2, *z*−1/2; (ii) −*x*, −*y*+2, −*z*+1; (iii) *x*−1/2, −*y*+3/2, *z*+1/2.
